# Major Insights in Dynamics of Host Response to SARS-CoV-2: Impacts and Challenges

**DOI:** 10.3389/fmicb.2021.637554

**Published:** 2021-08-25

**Authors:** Al Hakim, Md. Mahbub Hasan, Mahmudul Hasan, Syed Mohammad Lokman, Kazi Faizul Azim, Topu Raihan, Parveen Afroz Chowdhury, Abul Kalam Azad

**Affiliations:** ^1^Department of Genetic Engineering and Biotechnology, School of Life Sciences, Shahjalal University of Science and Technology, Sylhet, Bangladesh; ^2^Department of Genetic Engineering and Biotechnology, Faculty of Biological Sciences, University of Chittagong, Chittagong, Bangladesh; ^3^Institute of Pharmaceutical Science, School of Cancer and Pharmaceutical Sciences, King’s College London, Franklin-Wilkins Building, London, United Kingdom; ^4^Department of Pharmaceutical and Industrial Biotechnology, Sylhet Agricultural University, Sylhet, Bangladesh; ^5^Department of Microbial Biotechnology, Faculty of Biotechnology and Genetic Engineering, Sylhet Agricultural University, Sylhet, Bangladesh; ^6^Department of Dermatology, Sylhet Women’s Medical College, Sylhet, Bangladesh

**Keywords:** comorbidities, coronaviruses, dynamics, host response, SARS-CoV-2, COVID-19, SARS-CoV-2 variants, COVID-19 vaccine

## Abstract

The coronavirus disease 2019 (COVID-19), a pandemic declared by the World Health Organization on March 11, 2020, is caused by the infection of highly transmissible species of a novel coronavirus called severe acute respiratory syndrome coronavirus-2 (SARS-CoV-2). As of July 25, 2021, there are 194,372,584 cases and 4,167,937 deaths with high variability in clinical manifestations, disease burden, and post-disease complications among different people around the globe. Overall, COVID-19 is manifested as mild to moderate in almost 90% of the cases and only the rest 10% of the cases need hospitalization. However, patients with older age and those having different comorbidities have made worst the pandemic scenario. The variability of pathological consequences and clinical manifestations of COVID-19 is associated with differential host–SARS-CoV-2 interactions, which are influenced by the factors that originated from the SARS-CoV-2 and the host. These factors usually include the genomic attributes and virulent factors of the SARS-CoV-2, the burden of coinfection with other viruses and bacteria, age and gender of the individuals, different comorbidities, immune suppressions/deficiency, genotypes of major histocompatibility complex, and blood group antigens and antibodies. We herein retrieved and reviewed literatures from PubMed, Scopus, and Google relevant to clinical complications and pathogenesis of COVID-19 among people of different age, sex, and geographical locations; genomic characteristics of SARS-CoV-2 including its variants, host response under different variables, and comorbidities to summarize the dynamics of the host response to SARS-CoV-2 infection; and host response toward approved vaccines and treatment strategies against COVID-19. After reviewing a large number of published articles covering different aspects of host response to SARS-CoV-2, it is clear that one aspect from one region is not working with the scenario same to others, as studies have been done separately with a very small number of cases from a particular area/region of a country. Importantly, to combat such a pandemic as COVID-19, a conclusive understanding of the disease dynamics is required. This review emphasizes on the identification of the factors influencing the dynamics of host responses to SARS-CoV-2 and offers a future perspective to explore the molecular insights of COVID-19.

## Introduction

The coronavirus disease 2019 (COVID-19) caused by a novel coronavirus named severe acute respiratory syndrome-coronavirus-2 (SARS-CoV-2), first found in Wuhan, China, in December 2019, has become a global emergency. Consequently, on March 11, 2020, the World Health Organization (WHO) declared COVID-19 as a pandemic (WHO, 2020b). It has spread in 220 countries and regions, and as of July 25, 2021, 194,372,584 cases of COVID-19 and 4,167,937 deaths have been confirmed (WHO, 2020c).

**FIGURE 1:**
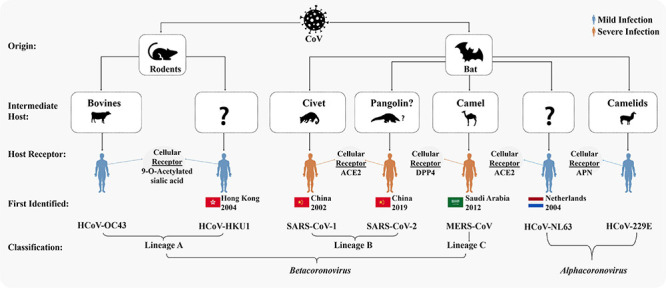
Origins and taxonomy of human coronaviruses (HCoVs). Severe acute respiratory syndrome coronavirus (SARS-CoV-1) and Middle East respiratory syndrome coronavirus (MERS-CoV) were transmitted to humans from bats by civet cats and dromedary camels, respectively. The severe acute respiratory syndrome coronavirus-2 (SARS-CoV-2) was likely transmitted to human from bats *via* an intermediate host pangolin (Andersen et al., 2020; Sun et al., 2020; Wu F. et al., 2020; Zhang T. et al., 2020; Zhou P. et al., 2020). Cellular receptors of all the seven HCoVs are mentioned. Among the seven HCoVs, HCoV-229E and HCoV-NL63 belong to *Alphacoronavirus*; HCoV-OC43 and HCoV-HKU1 belong to lineage A, SARS-CoV-1 and SARS-CoV-2 to lineage B, and MERS-CoV to lineage C of *Betacoronavirus*.

Coronaviruses are enveloped, single-stranded, positive-sense RNA viruses, first recognized in the 1960s, and cause respiratory tract infection (Tyrrell and Bynoe, 1966; Kahn and Mcintosh, 2005). Before COVID-19, six human coronaviruses (HCoVs) have been reported to cause respiratory diseases (Fung and Liu, 2019; Rabi et al., 2020). Among them, HCoV-229E and HCoV-NL63 belonging to *Alphacoronavirus* and HCoV-OC43 and HCoV-HKU1 belonging to *Betacoronavirus* generally cause mild to moderate upper respiratory tract illness, producing common cold in ∼15–30% of cases (Fung and Liu, 2019; Liu D.X. et al., 2020; Rabi et al., 2020). Other two β-coronaviruses, severe acute respiratory syndrome coronavirus (SARS-CoV-1) and Middle East respiratory syndrome coronavirus (MERS-CoV), are zoonotic and produced regional and global outbreaks, SARS and MERS in 2002–2003 and 2012, respectively (Fung and Liu, 2019). SARS-CoV-2, defined as a β-coronavirus (Figure 1), shares 80 and 50% genetic identity with SARS-CoV-1 (Kim et al., 2020a; Zhou P. et al., 2020) and MERS-CoV (Jiang et al., 2020a), respectively. However, SARS-CoV-2 shows similar clinical characteristics as SARS-CoV1 and MERS-CoV (Jiang et al., 2020a). Although the morbidity of COVID-19 is lower than that of SARS or MERS, COVID-19 is spreading in an alarming rate compared to either of them (Jiang et al., 2020c), and it has been confirmed that COVID-19 is transmitted from human to human (Phan et al., 2020). Based on the clinical characteristics, COVID-19 patients are classified as (i) mild, (ii) moderate, (iii) severe, and (iv) critical (Jin A. et al., 2020). Severe or critical patients need to be admitted in an intensive care unit (ICU). However, a large percentage (adult, 10.1–23.0%; children, 16.4–42.7%; and ∼50% of the patients with no symptoms during detection develop symptoms later) of infected individuals remain asymptomatic and serve as reservoirs and carriers (Tan et al., 2020). Comorbidities such as diabetes; hypertension; obesity; older age (greater than 60 years); cardiac, hepatic, and renal disorders; malignancy; coinfection; immunodeficiency; etc. not only increase the risk of sprouting severe illness but also enhance the risk of death (Sanyaolu et al., 2020). Currently, only supportive treatments are being given to COVID-19 patients, as no effective newly developed specific drug has been yet approved. However, to tackle the present crisis, researches are going on for understanding the epidemiology (Chen N. et al., 2020), pathogenicity (Hussain et al., 2020; Yuki et al., 2020), clinical characteristics (Chen T. et al., 2020; Huang C. et al., 2020), transmission dynamics (Kucharski et al., 2020), comorbidities as the risk factors (Sanyaolu et al., 2020; Wu C. et al., 2020), including its genomic variance and molecular insights (Cárdenas-Conejo et al., 2020; Shen et al., 2020; Wen et al., 2020), entrance into and interaction with the host cells as well as the replication (Shi J. et al., 2020; Zhou P. et al., 2020), and the immune response of the infected individuals (Cao, 2020; Chen G. et al., 2020; García, 2020; Li et al., 2020a; Tay et al., 2020). Furthermore, several vaccines against COVID-19 have been developed and approved by the WHO.^1^

Host response is an outcome of the host–microbe interaction, which provides information and makes the researchers understand the pathogenesis and biology of the pathogens as well as the host that are essential steps for managing and controlling the disease (Casadevall and Pirofski, 2000; Pirofski and Casadevall, 2020). Host response to SARS-CoV-2 is associated with the development of COVID-19 (Blanco-Melo et al., 2020). Proper understanding about the factors of the SARS-CoV-2 and the host associated with host response will explore the prevention and therapeutic implications of COVID-19. Several reviews have been done focusing on only one factor such as age, sex, immunity, and comorbidity (Callender et al., 2020; Gadi et al., 2020; Scully et al., 2020; Shah et al., 2020). However, no comprehensive review has been published on the dynamics of host response to SARS-CoV-2 considering all potential factors that originated from the causative agent and the exposed individual. The present review focuses on the aspects of insights in dynamics of host responses to SARS-CoV-2 in context of pathogenicity, genomic attributes of SARS-CoV-2, demographical and racial variance, age and gender, coinfection, comorbidities ranging from asymptomatic to critical clinical expositions, and vaccine and treatment strategies, and offers a future perspective to explore the molecular insights of COVID-19.

## Evolution and Genomic Attributes of SARS-CoV-2

**FIGURE 2:**
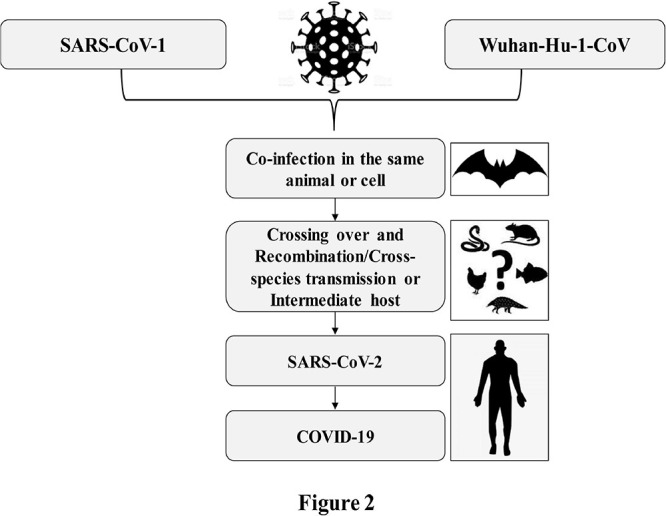
Crossing over due to coinfection and possible recombination of SARS-CoV-2 causing COVID-19.

All coronaviruses causing human diseases had originated from either bats or rodents (Fung and Liu, 2019). SARS-CoV-1 and MERS-CoV were transmitted directly to humans from civet cats and dromedary camels, respectively (Guan et al., 2003; Azhar et al., 2014). The complete genome sequences of SARS-CoV-2 isolated from diversified patients share more than 99.9% identity, indicating an immediate host shift of this virus to humans (Lu R. et al., 2020; Tang et al., 2020; Zhou P. et al., 2020). The whole genome of SARS-CoV-2 shares 96.3 and 91.2% identity to the genomes of bat SARS-related coronavirus, Bat-SARSr-CoV-RaTG13, and pangolin-CoV, respectively (Lam et al., 2020; Li X. et al., 2020; Lu R. et al., 2020; Tang et al., 2020; Zhou P. et al., 2020). Furthermore, the SARS-related coronaviruses possess spike protein having a variable receptor-binding domain (RBD), which binds to angiotensin-converting enzyme-2 (ACE2) receptors available in the lungs, heart, kidney, and gastrointestinal tract to facilitate the entry of the virus into the target cells (Ksiazek et al., 2003). The RBD sequence of SARS-CoV-2 is very close (99%) to that of a pangolin-CoV (Lam et al., 2020; Tang et al., 2020) and appears to be a mutated version of Bat-SARSr-CoV-RaTG13 (Andersen et al., 2020). Based on these findings, it is, therefore, believed that the SARS-CoV-2 with novel phenotypes has originated through a recombination of more distant CoVs (Andersen et al., 2020; Li X. et al., 2020). The close vicinity of animals of different species in a wild animal may enhance the plausible cross-species spillover infections for genetic recombination between more distant CoVs as illustrated in Figure 2. Genetic recombination is one of the important strategic evolutionary processes in the emergence of CoVs (Graham and Baric, 2010; Rehman et al., 2020). CoVs generally originated from zoonotic transmissions with intermediate host species between the bat reservoirs and humans (Lin et al., 2017; Banerjee et al., 2019; Cui et al., 2019). Therefore, the high prevalence of CoVs with large genetic diversity, frequent genome recombination, and increased human–animal interface behavior might have facilitated the emergence of novel CoVs from time to time in humans due to occasional spillover and recurrent cross-species infection events.

The worldwide databases including the National Center for Biotechnology Information (NCBI), the Global Initiative on Sharing All Influenza Data (GISAID), and the China National Center for Bioinformation 2019 Novel Coronavirus Resource (2019nCoVR) have been enriched with thousands of complete genome sequences of the SARS-CoV-2 from different parts of the world. By analyzing genome sequences of the SARS-CoV-2 retrieved from GSAID, it has been proposed that that the evolution of this virus is not specific to any particular country or territory but rather specific to a patient or ethnic group (Hoque et al., 2020). As the phylogenetic analysis of the genome sequences of SARS-CoV-2 revealed a common ancestor for the bats’ CoV and the human SARS-CoV-2, the global researchers classify the SARS-CoV-2 as a SARS-like virus (Andersen et al., 2020; Zhang L. et al., 2020). On the contrary, the genome sequences of pangolin-CoVs share 85.5 to 92.4% similarity to SARS-CoV-2 (Lam et al., 2020). The common ancestor for the bats’ CoV and human SARS-CoV-2 and the higher similarity of pangolin-CoVs to SARS-CoV-2 lead researchers to speculate that this novel virus might have transmitted to human from bats *via* an intermediate host pangolin through host jump (Andersen et al., 2020; Wu F. et al., 2020; Zhang T. et al., 2020; Zhou P. et al., 2020). However, presently accessible evidences are not sufficient enough to make a conclusive inference that the SARS-CoV-2 was directly transmitted from bats to humans or indirectly through an intermediate host, pangolin. Currently available data rather may explain the origin of SARS-CoV-2 either by a natural selection in humans following zoonotic transfer or that in an animal host before zoonotic transfer.

On the eve of the current pandemic, a phylogenetic network analysis of 160 SARS-CoV-2 complete genomes revealed three variants, namely, A, B, and C, distinguished by amino acid changes, which are circulating in different parts of the world, with A being the ancestral type according to the bat out group coronavirus (Forster et al., 2020). The A and C types are predominantly found in Europeans and Americans, and the B type is the most common in East Asians (Forster et al., 2020). While the B type is derived from type A by a synonymous mutation T8782C and a non-synonymous mutation C28144T, the C type is derived from type B by a non-synonymous mutation G26144T (Forster et al., 2020). However, variation in host response or pathogenicity is not yet established to these three variants. In another study, an analysis of 103 complete genomes of SARS-CoV-2 revealed that two major lineages, namely, L and S of SARS-CoV-2, well-defined by two different SNPs, are spreading around the world (Tang et al., 2020). The L lineage was found to be more prevalent (∼70%) than the S lineage (∼30%) in the small sample size (Tang et al., 2020).

During the current pandemic, the SARS-CoV-2 has adapted to their hosts through mutations in their genome, resulting in new variants that are expected to show different traits than the ancestral strains (Aleem et al., 2021). Several types of SARS-CoV-2 genetic variants are already reported to circulate around the world. Currently, four of them are considered as variants of concern (VOC). Among the VOC, the Alpha/501Y.V1/B.1.1.7 variant was reported in the United Kingdom in the late December 2020 (Volz et al., 2021), the Beta/501Y.V2/B.1.351 was reported in South Africa in the middle of December 2020 (Tegally et al., 2021), the Gamma/501Y.V3/P.1 was reported in Brazil in early January 2021 (Sabino et al., 2021), and the Delta/G/452R.V3/B.1.617 was identified in India in October 2020 (Planas et al., 2021). The VOC can increase transmissibility or virulence or escape neutralization and may impact the effectiveness of vaccines (discussed in the later section). Besides, the WHO and the Centers for Disease Control and Prevention (CDC) consider some as variants of interest (VOI), namely, B.1.525/484K.V3 (Eta), B.1.526/253G.V1 (Lota), B.1.617.1/452R.V3 (Kappa), C.37/452Q.V1 (Lambda), B.1.427/B.1.429/452R.V1 (Epsilon), P.3/1092K.V1 (Theta), B.1.617.3, and P.2/484K.V2 (Zeta) (Aleem et al., 2021).^2^ The VOI are reported from some specific countries. The genetic changes in VOI are considered to affect viral transmissibility, disease severity, and immune and diagnostic evasion.

**FIGURE 3:**
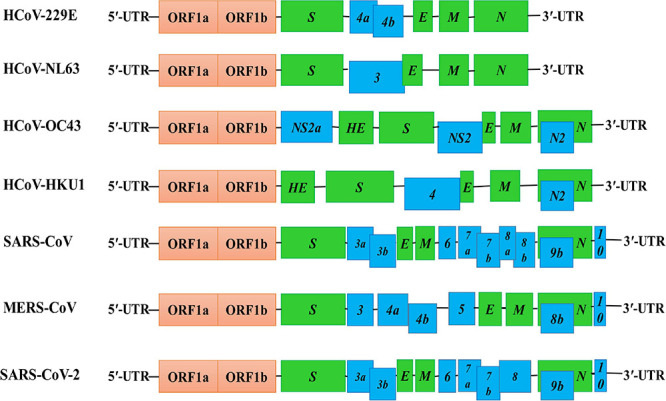
Genome structures of SARS-CoV-2 and other HCoVs. The schematic diagram shows the genome structure of seven HCoVs [the non-structural proteins’ (nsps) and open reading frames’ (orfs) lengths are not drawn in scale]. The 5′-UTR and 3′-UTR are indicated. The polyprotein with open reading frame 1a (ORF1a) and ORF1b are shown as red boxes. The genes encoding structural proteins spike glycoprotein (S), envelope (E), membrane (M), nucleocapsid (N), and hemagglutinin-esterase (HE) are indicated as green boxes. The genes encoding accessory proteins such as ORF 3a/b, 4a/b, 5, 6, 7a/b, 8a/b, 9b, and 10 are indicated as blue boxes.

Like other coronaviruses (Fung and Liu, 2019), the genome of the SARS-CoV-2 is a positively sensed, non-segmented, single-stranded RNA having a size of ∼30 kb (range: 29.8–29.9 kb) (Khailany et al., 2020). The genomic RNA being 5′-capped and 3′-polyadenylated encodes several open reading frames (ORFs). Similar to other HCoVs (Fung and Liu, 2019), the genome of SARS-CoV-2 encodes invariant gene order 5′-replicase polyprotein-spike (S)-envelope (E)-membrane (M)-nucleocapsid (N)-3′, with seven small ORFs (encoding accessory proteins) scattered among the structural genes (Figure 3). However, the genomic positions of the ORFs for the four structural proteins S, E, M, and N among betacoronaviruses are different as depicted in Figure 3. The small ORFs encoding accessory proteins are ORF3a, ORF6, ORF7a, ORF7b, ORF8, and ORF10 located in the 3′ region. The first ORF in the 5′ region encodes *orf1ab*, the largest gene in SARS-CoV-2, which encodes the replicase polyprotein (pp1ab) and 15 non-structural proteins (nsps) (Hoque et al., 2020; Shereen et al., 2020). Noticeable variations between SARS-CoV-1 and SARS-CoV-2 such as structural compositions and the number of amino acids in proteins encoded by ORF8 and ORF3 in SARS-CoV-2 have been reported by several studies (Figure 3; Hoque et al., 2020; Shereen et al., 2020; Wu A. et al., 2020). The genomic and protein identity of ORF8 in SARS-CoV-2 bears a strong identity with that in both bat and pangolin coronaviruses (Mohammad et al., 2020) and might be a potential recombination site (Lau et al., 2015) and involved in increased infectivity of SARS-CoV-2 (Sicari et al., 2020). The ORF3 may be involved in the attenuation of cellular protein synthesis (Bouhaddou et al., 2020; Sicari et al., 2020; Stukalov et al., 2020). The reduced apoptosis-mediated antiviral defense by the ORF3a of SARS-CoV-2 in infected cells might be associated with mild or even asymptomatic infection during early stages, and at this period, the virus may spread more widely (Ren et al., 2020). The SARS-CoV-2 ORF3b is a potent inhibitor of human IFN-I activation, and its antagonistic activity is significantly higher than that of SARS-CoV-1 ORF3b, which, in turn, affects the host response to SARS-CoV-2 (Konno et al., 2020). Hosts respond to SARS-CoV-2 through generation of ORF8 and ORF3b antibodies in early and late stages and, thus, can be used as serological markers (Hachim et al., 2020). However, more studies are necessary for the clear understanding of the mechanism of *ORF8* and *ORF3* gene products, which would be important for therapeutic intervention against SARS-CoV-2.

## Pathogenesis of COVID-19 and Host Response

Pathogenesis is a process of disease development following an infection. Viral pathogenic mechanisms comprise (1) attaching of virus to the host receptor at the route of entry, (2) viral replication at the entry site, (3) viral spread to target organs, and (4) shedding of virus into the environment (Baron et al., 1996). Factors affecting viral pathogenesis include (1) accessibility of virus to tissue, (2) cell susceptibility to virus multiplication, and (3) virus susceptibility to host defense, and thus, pathogenesis depends on the viral intrinsic factors such as its genomics and virulence factors, the state of host health, age, gender, and stress or comorbidities.

### Transmission of SARS-CoV-2 to Humans

The routes of entry of SARS-CoV-2 to human include the nose, mouth, and eyes (Li H. et al., 2020). The virus can be spread when an individual touches the mucosal membranes of the nose, mouth, and eye after touching the surface of an object contaminated with SARS-CoV-2 (WHO, 2020e). SARS-CoV-2 is transmitted predominantly *via* droplet of saliva and discharges from the nose of an infected person; by direct body contact with the infected individual; and through airborne, fomite, fecal–oral, blood-borne, and animal-to-human transmission (Vivanti et al., 2020; Wang D. et al., 2020; Wang et al., 2020a). Transplacental transmission of SARS-CoV-2 from mother to fetus has been confirmed by comprehensive virological and pathological investigations (Vivanti et al., 2020). Maternal viremia with high viral load as well as neonatal viremia caused by SARS-CoV-2 has been confirmed by histological and immunochemistry experiments, and neurological manifestations were developed in the neonate as was observed in adult patients (Vivanti et al., 2020).

### Entrance and Viral Loads of SARS-CoV-2 Are Associated With the Dynamics of Host Response

**FIGURE 4:**
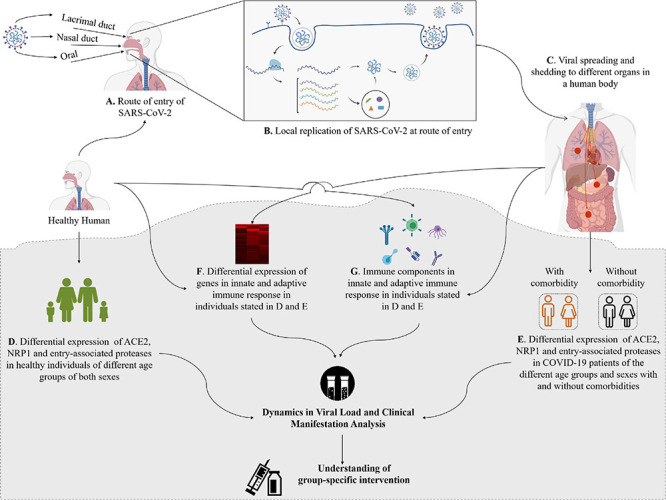
Entry and shedding of SARS-CoV-2 into humans **(A–C)**, and future perspectives for molecular insights in dynamics of host response to SARS-CoV-2 [lower shaded part **(D–G)**].

The spike glycoprotein S of HCoVs binds to the cell surface enzymes as receptors (Figure 1), mediating membrane fusion and viral entry into the host cells (Lim et al., 2016; Fung and Liu, 2019). The cleavage of S protein in different HCoVs into S1 and S2, which causes binding and membrane fusion, respectively, is mediated by one or more diverse host proteases (Fung and Liu, 2019; Yan et al., 2020). The S1 subunit of S protein of SARS-CoV-1 and SARS-CoV-2 having the RBD shares the ACE2 as the receptor expressed on the surface of the target cells in different human organs, which are vulnerable to SARS-CoV-2 (Lim et al., 2016; Chen Y. et al., 2020; Hoffmann et al., 2020; Ou J. et al., 2020; Rahman et al., 2020; Walls et al., 2020; Yan et al., 2020; Zou X. et al., 2020). However, the binding affinity of the RBD of SARS-CoV-2 for ACE2 is ∼10–20 times higher than that of SARS-CoV-1, which could be a reason for the higher infectivity of SARS-CoV-2 (Shang et al., 2020; Wrapp et al., 2020). Some amino acid residual variations in the interfaces of SARS-CoV-2-RBD and SARS-CoV-1-RBD and a furin-like cleavage site present in the S protein of the SARS-Co-V-2 but not in SARS-like CoVs may strengthen the interaction of SARS-CoV-2-RBD to ACE2 to increase the biding affinity (Coutard et al., 2020; Yan et al., 2020). Furthermore, neuropilin-1 (NRP1) expressed profusely in pulmonary and olfactory endothelial cells binds furin-cleaved substrates and facilitates entry and infectivity of SARS-CoV-2 through co-expression with ACE2 and the transmembrane protease serine 2 (TMPRSS2) (Cantuti-Castelvetri et al., 2020). The TMPRSS2 leading to cleavage of ACE2 and activation of the S protein mediates coronavirus entry into host cells (Hoffmann et al., 2020). Both ACE2 and TMPRSS2 are highly expressed in nasal goblet and ciliated cells in healthy persons (Sungnak et al., 2020). A higher expression of the ACE2 and viral entry-associated proteases in cells of different organs, high ACE2-binding affinity of the RBD, preactivation of the S protein, and the hidden RBD in the S protein might have permitted SARS-CoV-2 to uphold efficient cell entry and made the cells possible reservoirs with higher viral load for dissemination within and between individuals (Shang et al., 2020; Sungnak et al., 2020; Zou X. et al., 2020). A study shows that the interferon-responsive genes including ACE2 are increased by high viral load, while transcripts for B cell-specific proteins and neutrophil chemokines are elevated with lower viral load (Lieberman et al., 2020). Variation in viral load and efficient cell entry will be associated with the SARS-CoV-2 pathogenicity and the host response dynamics *via* innate and adaptive immunity (Chen and Li, 2020; Shang et al., 2020). However, Sungnak et al. (2020) investigated the expression profiles of ACE2 and the possible SARS-CoV-2 entry-associated proteases in healthy individuals only, and a detailed study with large number of samples for comparing the expression profiles of these proteins in infected and non-infected individuals is still absent. Therefore, researchers need to focus on differential expression analysis of (i) ACE2 and SARS-CoV-2 entry-associated proteases in healthy persons of different age groups of male and female and in COVID-19 patients of the same age groups and sex with and without different comorbidities and (ii) genes and immune components of innate and adaptive immune response (Figure 4). This will be important for a detailed understanding about the pathogenicity of SARS-CoV-2 and host response dynamics in patients of different age and sex with/without comorbidities and, thus, contribute to understanding and designing intervention, vaccination, and epidemiological control of COVID-19.

Viral load and shed might be used as a potential marker for determining the host response, disease severity, and prognosis, which are correlated with the infectivity, morbidity, and mortality (Chen and Li, 2020; Liu Y. et al., 2020). The viral load of SARS-CoV-2 peaks at the time of infection onset in contrast with that of SARS-CoV-1, which peaks at around 10 days after symptom onset, and that of MERS-CoV, which peaks at the second week after symptom onset (To et al., 2020). The viral RNA has been detected in nasopharyngeal and oropharyngeal swab specimens, sputum, bronchoalveolar lavage fluid, saliva, whole blood, serum, breastmilk, placental samples, stool, and urine (Groß et al., 2020; Huang Y. et al., 2020; Li H. et al., 2020; Penfield et al., 2020; Wang X. et al., 2020; Yu X. et al., 2020), indicating that the SARS-CoV-2 can transmit to other organs or systems of the body after getting entrance by any of the three routes or sharing the routes aforementioned (Li H. et al., 2020). However, while one type of sample turns to SARS-CoV-2 negative, another type of sample may become positive (Table 1), and the viral load in different samples peaks at different time after symptom or disease onset (Pan et al., 2020b; Wu C. et al., 2020; Zheng S. et al., 2020). Viral shedding is observed in a variety of tissues in severely ill patients for 20–40 days after onset of disease with higher level of IgM, while that is restricted to the respiratory tract for 10 days after onset with lower IgM (Wang Y. et al., 2020). However, viral shedding begins approximately 2–3 days prior to the onset of symptoms, and it can persist for up to 8 days in mild cases and for longer periods in severe cases (Li and Xu, 2009; He X. et al., 2020; Wölfel et al., 2020). Therefore, the appearance of clinical manifestation and its type might be associated with the initial route of entry, intra-transmission pathway, and the viral load. The persistence of viral load for SARS-CoV-2 is sex dependent and significantly longer in male than in female, which is consistent with the severity of the disease caused by SARS-CoV-1 and MERS-CoV (Karlberg et al., 2004; Alghamdi et al., 2014; Zheng S. et al., 2020). Furthermore, asymptomatic patients are reported to have viral load similar to that in the symptomatic patients and serve as reservoir for transmission (Walsh et al., 2020; Zou L. et al., 2020). These findings suggest that reducing the viral loads through clinical management may play an important role for preventing the spread of the virus.

**TABLE 1:** Viral load in different samples from COVID-19 patients.

**Different samples**	**Time required for viral peak**	**Viral load (Ct/copies of viral particle)**	**References**
Nasal swab	Day 3 post-onset	1⋅69 × 10^5^ copies/ml	Pan et al., 2020b
Throat swab	Days 3–15 post-onset	7⋅99 × 10^4^ copies/ml	Pan et al., 2020b
Sputum/ETA	Days 3–15 post-onset	7⋅52 × 10^5^ copies/ml	Pan et al., 2020b
Feces	Days 0–11 after onset	550 to 1⋅21 × 10^5^ copies/ml	Pan et al., 2020b
Saliva	1st week of symptom onset	5.2 log_10_ copies/ml (158,489)	To et al., 2020
Plasma	–	2.4 log_10_ RNA copies/ml (25,119)	Fajnzylber et al., 2020
Urine	Day 11 post-onset	2.09 ± 0.85 log_10_ copies/ml (126)	Jeong et al., 2020

## Clinical Manifestations and Complications

Humans of both sexes of all ages are susceptible to SARS-CoV-2 with mild disease in children and adolescent (Chen N. et al., 2020; Götzinger et al., 2020). Several studies show that most of the children infected with SARS-CoV-2 are asymptomatic or have mild symptoms, and about 11% of them need hospitalization (Bialek et al., 2020; Dong et al., 2020; Götzinger et al., 2020; Parri et al., 2020; Wang D. et al., 2020). The symptoms of COVID-19 commonly include fever, dry cough, and tiredness; uncommonly headache, body pain, sore throat, diarrhea, loss of taste or smell, conjunctivitis, and rashes on the skin or discoloration of fingers; and severely shortness of breath, rapid falling of oxygen concentration, chest pain, and loss of speech or movement (Guan et al., 2020; Mao R. et al., 2020; Mehta et al., 2020; Sanyaolu et al., 2020). The frequency of clinical manifestations of COVID-19 hospitalized patients includes fever (70–90%); dry cough (60–86%); shortness of breath (53–80%); fatigue (38%); myalgias (15–44%); gastrointestinal symptoms, i.e., nausea/vomiting, diarrhea, and loss of appetite (15–39%); headache and weakness (25%); and rhinorrhea (7%) (Guan et al., 2020; Mao R. et al., 2020). Based on these clinical characteristics, COVID-19 patients are classified as (i) mild, (ii) moderate, (iii) severe, and (iv) critical (Jin A. et al., 2020). Severe or critical patients need to be admitted in ICU. However, a large percentage of infected individuals remain asymptomatic and serve as reservoirs and carriers (Tan et al., 2020). The percentage of asymptomatic infections varies based on age, gender, and regions (He J. et al., 2020). The symptoms of COVID-19 infection appear after a mean incubation period of approximately 5 (2–7) days (Guan et al., 2020; Lauer et al., 2020). The length of period from the appearance of the first symptoms to death ranged from 6 to 41 days with a median of 14 days (Wang et al., 2020a). This period is dependent on the age and status of the patient’s immune system. It tended to be shorter among patients aged 70 years or more (ranged usually 6–19 days) than those aged less than 70 years (10–41 days) (Wang et al., 2020a). The risk of death depends on age, sex, comorbidities, and severity of the disease (Wu C. et al., 2020), which are discussed in the forthcoming sections. Organs that are the most affected and generate some clinical complications include the lungs, followed by the heart, kidneys, liver, brain, and gastrointestinal system (Zhou F. et al., 2020).

### Respiratory Complications and Hypoxia

Respiratory failure caused by SARS-CoV-2 infection, defined as the severe acute respiratory infection (SARI) by the WHO (2020a), is one of the critical host responses (Lechowicz et al., 2020). In some cases, SARI could lead to acute respiratory distress syndrome (ARDS), which may cause more complicated situation by death or sepsis-related complications (Chen N. et al., 2020; Li Q. et al., 2020; Wang D. et al., 2020). The ARDS developed in COVID-19 patients increases the mortality up to 49%, and age, neutrophilia, elevated lactate dehydrogenase (LDH), and D-dimer have been identified as the risk factors for the development of ARDS (Wu C. et al., 2020). The consequence of the direct invasion of SARS-CoV-2 to pneumocytes is the reduced expression of ACE2 and cleavage of angiotensin II to develop angiotensin (Chen N. et al., 2020; Ellinghaus et al., 2020; Huang C. et al., 2020; Lechowicz et al., 2020; Li Q. et al., 2020; Wang D. et al., 2020). The intact angiotensin II raises vascular permeability of the lung tissue leading to ARDS by stimulating pro-inflammatory responses (Bornstein et al., 2020). The acute condition of pneumonia in COVID-19 can be detected by shortened breath, respiratory rate >30/min, and oxygen saturation <90% (Huang C. et al., 2020; Wu and Mcgoogan, 2020). During the COVID-19 pandemic, pulmonary fibrosis has been identified as one of the major complications caused by damaging lung tissue and scarring in infected patients (Haschek and Witschi, 1979; Wang J. et al., 2020). In pulmonary fibrosis, irregular solid nodules in pulmonary region had been identified in 12.7% patients, fibrous strips had been reported for 17.5% patients; nodules and strips were found enlarged in approximately 85% cases during disease progression, and patchy GGO were detected as the most frequent change (Grasselli et al., 2020; Pan et al., 2020a; Shi H. et al., 2020; Wang J. et al., 2020).

Different cytokines and growth factors have been reported to be released during the period of lung damage including pulmonary fibrosis. For example, tumor necrosis factor, monocyte chemoattractant protein (MCP), platelet-derived growth factor, transforming growth factor-1, interleukin-1b (IL-1b), fibroblast growth factor, and interleukin-6 (IL-6) are reported to be over expressed and released during the severe damage in the lung cells (Razzaque and Taguchi, 2003; Grimminger et al., 2015; Jin Y. et al., 2020). Severe COVID-19 patients show an elevated level of pro-inflammatory cytokines including IL-2, IL-7, IL-10, granulocyte colony-stimulating factor, γ-interferon-induced protein 10, MCP, macrophage inflammatory proteins, and tumor necrosis factor-α (TNF-α), and the inflammatory cascade may lead to a cytokine storm, which is a key factor for driving both ARDS and extra-pulmonary organ failure (Li and Xu, 2009; Huang C. et al., 2020). Several studies suggested that the abovementioned growth factors and cytokines are abundant in the serum, and the cytokine profile in the older COVID-19 patients could make them more susceptible to pulmonary fibrosis and other respiratory complications (Nile et al., 2020; Xiong et al., 2020; Yuki et al., 2020).

In COVID-19 patients, hypoxia is an extremely important factor for causing multi-organ dysfunction or damage including acute cardiac injury (ACI) (Nan et al., 2020), acute kidney injury (AKI) (Del Vecchio and Locatelli, 2020), and neurological dysfunction (Parry et al., 2020). COVID-19 patients with ACI are more susceptible to ARDS compared with COVID-19 patients without ACI (Chen T. et al., 2020; Shi S. et al., 2020). Hypoxia in severe SARS-CoV-2 infection involves the hypoxia-inducible factor system, which might have influenced the inflammatory response and outcome in the kidney and lung (Del Vecchio and Locatelli, 2020). Acute and prolonged hypoxia may result in hypoxic ischemic encephalopathy and demyelination or microhemorrhages, respectively (Parry et al., 2020).

### Neurological Complications

Similar to SARS and MERS patients, neurotropism is one of the most common features in COVID-19 patients (Li et al., 2012; Li Y.C. et al., 2020). SARS-CoV-2 may invade the human body either through immune-mediated pathways or a direct attack in the nervous system (Beghi et al., 2020). A retrospective study on 214 COVID-19 patients in Wuhan, China, reported that 36.4% of patients had neurological disorders imparting clinical manifestations in the central nervous system (24.8%; dizziness, headache, impaired consciousness, acute cerebrovascular disease, ataxia, and seizure), peripheral nervous system (8.9%; taste, smell, and vision impairment and nerve pain), and skeletal muscle (10.7%) (Mao L. et al., 2020). A meta-analysis with 59,254 confirmed COVID-19 cases found that 12% of patients suffered from headache (Borges Do Nascimento et al., 2020; Valderrama et al., 2020). Olfactory and gustatory dysfunctions and headaches are very common symptoms in mild cases, whereas impaired consciousness, delirium, and muscle pain are common in cases of severe COVID-19 patients (Lechien et al., 2020; Orsucci et al., 2020). Stroke (ischemic, hemorrhagic, and coagulopathy), sinus venous thrombosis, cerebral hemorrhage, encephalopathy, altered mental status, meningitis, encephalitis, febrile seizures, acute hemorrhagic necrotizing encephalopathy, acute disseminated encephalomyelitis, myelitis, myasthenia gravis, Miller Fisher syndrome, Guillain-Barre syndrome, and polyneuritis cranialis have been reported in several studies with COVID-19 (Aghagoli et al., 2020; Baig et al., 2020; Hirano and Murakami, 2020; Toscano et al., 2020).

### Gastrointestinal Complications

Gastrointestinal (GI) complications are associated with some COVID-19 patients. It is reported that approximately 2–10% of COVID-19 patients had accompanied GI symptoms such as diarrhea, abdominal pain, and vomiting (Chen N. et al., 2020). A systematic review and meta-analysis of 4,805 COVID-19 patients reported that the incidence of diarrhea and vomiting was 7.4 and 4.6%, respectively (Parasa et al., 2020). As aforementioned, the ACE2 receptor is significantly expressed in the epithelial cells of GI (Xiao et al., 2020; Zou X. et al., 2020), and the RNA of SARS-CoV-2 was detected in 69% of 842 stool samples (Zheng S. et al., 2020), indicating direct viral invasion in the GI of COVID-19 patients (Ong et al., 2020; Zhou Z. et al., 2020). These findings suggest that SARS-CoV-2 can be transmitted through the fecal–oral route.

## Host’s Factors Contributing to Dynamics of Response to SARS-CoV-2

### Age and Sex-Dependent Host Response

**FIGURE 5:**
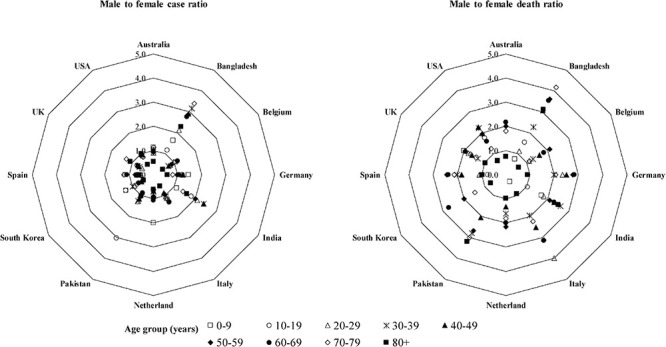
Male-to-female case and death ratios at different age groups. The data were extracted from the following sources: Australia (https://www.health.gov.au/news/health-alerts/novel-coronavirus-2019-ncov-health-alert/coronavirus-covid-19-current-situation-and-case-numbers#cases-and-deaths-by-), Bangladesh (https://www.who.int/docs/default-source/searo/bangladesh/covid-19-who-bangladesh-situation-reports/who-covid-19-update-32-20201005.pdf?sfvrsn=9ac4 1c99_2), Belgium (https://epistat.wiv-isp.be/covid/), Germany [https://www.rki.de/EN/Content/infections/epidemiology/outbreaks/COVID-19/COVID19.html, https://www.statista.com/statistics/1105512/coronavirus-covid-19-deaths-by-gender-germany/(Survey time period as of October 7, 2020)], India (Gupta et al., 2020; https://m.hindustantimes.com//india-news/), Italy (https://www.epicentro.iss.it/coronavirus/bollettino/Bollettino-sorveglianza-integrata-COVID-19_27-ottobre-2020.pdf, https://dc-covid.site.ined.fr/en/data/italy/), Netherlands (https://data.rivm.nl/covid-19/), Pakistan (https://globalhealth5050.org/the-sex-gender-and-covid-19-project/), South Korea (http://www.kdca.go.kr/cdc_eng/), Spain (https://www.isciii.es/QueHacemos/Servicios/VigilanciaSaludPublicaRENAVE/EnfermedadesTransmisibles/Paginas/-COVID-19.-Informes-previos.aspx), United Kingdom (https://www.statista.com/statistics/1115083/coronavirus-cases-in-england-by-age-and-gender/, https://dc-covid.site.ined.fr/en/data/pooled-datafiles/), and United States (https://covid.cdc.gov/covid-data-tracker/#demographics).

In most of the regions of the world including China, Italy, United States, and Australia, the severity of COVID-19 is more in men than women (COVID Data, 2020). A meta-analysis with published literatures revealed that males had a three-fold higher risk to mortality compared to females (Nasiri et al., 2020). The death ratio in male to female with COVID-19 is more than double in Italy and China (Global Health, 2020; Jin A. et al., 2020). An analysis of available data from 12 countries revealed that this ratio may be up to four-fold for some countries (Figure 5). In the total death, male is accounted 60–70%. Interestingly, COVID-19 cases in males and females are similar in almost countries, and in some countries, female cases are even more than male cases in certain age groups (Figure 5). However, the case ratio between males and females in Bangladesh and India is 1.5–3.5 and 1.5–2.5, respectively (Figure 5). The reason may be the more involvement of males to all outdoor activities compared to females in both countries.

**FIGURE 6:**
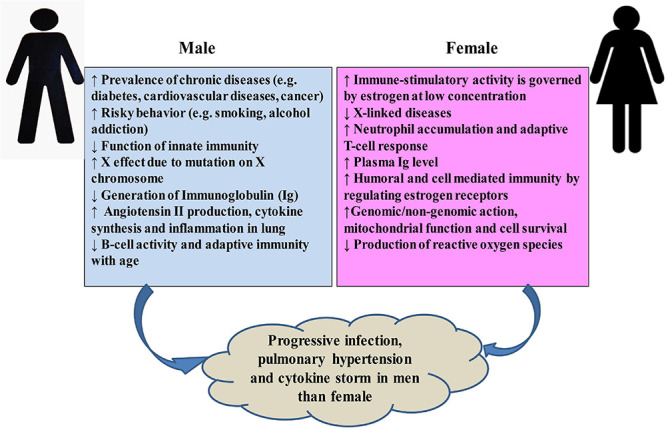
Possible sex-specific phenomena that may affect host response and lead to the progression of COVID-19 and post-complications.

The higher mortality rate of COVID-19 in males can be attributed to several factors (Figure 6). Men are more prone to various chronic diseases such as diabetes (Wannamethee et al., 2007), cardiac diseases (Weidner, 2000), and cancer (Nicholas, 2000). Generally, males are characterized by much risky behavior like smoking or alcohol addiction (Ritchie and Roser, 2013). All these severe risk factors may account for limited lifespan among SARS-CoV-2-infected patients (Cai, 2020; Guan et al., 2020; Huang C. et al., 2020; Zhang J.J. et al., 2020; Zhou F. et al., 2020). Variations in number and functions such as cellular metabolism, blood clotting, development, and induction of various diseases by genes in X and Y chromosomes may be responsible for some of the sex-biased mortality rate (Simon, 2010). Again, tissues of the immune system are significantly affected by the hormonal secretion during the activation period in males and females (Gaumond et al., 2002). While testosterone suppresses innate immunity, estrogen shows immune-stimulatory activity at a low concentration by stimulating adaptive T-cell response (Malkin et al., 2004; Straub, 2007; Rettew et al., 2008; Robinson et al., 2014; Klein and Flanagan, 2016). Women synthesize higher levels of estrogen and regulate the activation/expression of estrogen receptors on their immune cells, i.e., T and B-lymphocytes, macrophages, mast cells, dendritic cells, and NK (Nilsson et al., 2001), which in turn helps to confer stronger humoral and cell-mediated immunity (Bird et al., 2008; Kovats, 2015). The protective role of estrogen signaling against influenza virus infection in nasal epithelial cells has been demonstrated (Peretz et al., 2016). Researchers in China found that women with high estrogen levels tended to have less severe effects of COVID-19 than women with low levels of this hormone (Ding et al., 2020). Among the circulating estrogens, β-estradiol (E2) is the most abundant, which is produced by theca and granulosa cells. E2 induces the expression of endothelial nitric oxide synthase, a potent vasodilator, and reduces the production of reactive oxygen species, and thus increases cell survival (Iorga et al., 2017). Researchers in Stony Brook, New York, have already investigated the efficacy of short-term estrogen replacement therapy both in men and women among COVID-19 patients in the ICU (COVID Data, 2020), although it is still controversial (Menazza and Murphy, 2016; Iorga et al., 2017). It emphasizes the need for more detailed studies of genes and hormones in males and females, which may lead to variation in responses to COVID-19.

The age of a COVID-19 patient has been encountered as the greatest risk factor (Shim et al., 2020; Verity et al., 2020). The morbidity and the mortality of COVID-19 are higher in older people. Data from different countries interpreted in Figure 5 and in other studies indicated that about 80% of deaths in COVID-19 are observed among adults aged ≥65 years, with the highest percentage of severe outcomes among persons aged ≥80 years (Guan et al., 2020; Onder et al., 2020). During aging, a systemic, chronic, and low-grade proinflamatory response called inflammaging develops, which is characterized by increases in systemic proinflammatory cytokine levels, namely, IL-1b, IL-6, and TNF-αa, and contributes to the pathogenesis of pathogens (Franceschi et al., 2018). The innate immune system may become deteriorated with aging, and the changes may impact age-related vulnerability to COVID-19, including altered cytokine response to immune activators, impaired phagocytosis by macrophages and dendritic cells, and altered Toll-like receptor expression (Oh et al., 2019). Furthermore, age leads to a decline in adaptive immune function and an increase in proinflammatory activity (Márquez et al., 2020). Therefore, the higher mortality of COVID-19 in aged people could be the age-related decline in immune and inflammatory responses, which, in turn, can lead to a cytokine storm (Wu C. et al., 2020), and COVID-19 is usually mild or asymptomatic in young people with a healthy immune system (Lu X. et al., 2020). Moreover, all physiological systems and cellular processes decline with age (López-Otín et al., 2013), and comorbid conditions such as hypertension, coronary heart disease, diabetes, and others steadily increase with the aging and raise the risk of COVID-19 (Dugoff et al., 2014; Zhou F. et al., 2020).

### Blood Group-Specific Host Response

The correlation between ABO blood type and cancers, heart disease, and other infections has already been reported (Cheng et al., 2005; Liumbruno and Franchini, 2013; Harris and Larocque, 2016; Batool et al., 2017; Zhao et al., 2020; Zietz and Tatonetti, 2020). Zhao et al. (2020) compared ABO blood group distribution in 2,173 COVID-19-positive patients from three different hospitals in Wuhan, China. Blood group A was associated with a higher risk for SARS-CoV-2 infections and mortality compared to non-A blood groups, whereas blood group O was associated with a lower risk (Zhao et al., 2020). Another study with a meta-analysis including 31,100 patients found the similar results reporting blood group AB associated with higher risk and blood group O associated with lower risk of COVID-19 severity (Wu B.B. et al., 2020). Due to the sequence similarity (Lu R. et al., 2020) and similar receptor-binding patterns of SARS-CoV-1 and SARS-CoV-2 (Li et al., 2003; Hoffmann et al., 2020; Wan et al., 2020), the higher susceptibility of blood type A and lower susceptibility of blood type O for COVID-19 could be attributed to the presence of natural antibodies in blood (Gérard et al., 2020). The natural anti-A or -B antibodies in blood group O could bind to the S protein and block its interaction with ACE2. The anti-A from blood group O is more protective than anti-A from blood group B (Gérard et al., 2020).

### Human Leukocyte Antigen Variation and COVID-19 Outcomes

With more than 25,000 alleles for 45 human leukocyte antigen (HLA), loci having the highest polymorphic regions of human genomes are located within major histocompatibility complex region (Robinson et al., 2020). The diversity of nucleotide variation around the globe is significantly correlated with geographic locations (Buhler and Sanchez-Mazas, 2011). These HLA alleles are critical for determining host antiviral response as they are involved in the processing and presenting viral peptides to T-cells (Kumar et al., 2020). So, the polymorphic nature of HLA alleles in people of different regions might cause different outcomes of pathogenic infections of SARS-CoV-2 (Tian et al., 2017). A few studies have been conducted to deduce the role of HLA on SARS-CoV-2 pathogenicity (Table 2). A computational study analyzed the binding affinity of 145 MHC class 1 (HLA-A, -B, and -C) allelic products against all SARS-CoV-2 peptides (Nguyen et al., 2020). According to this study, HLA-A^∗^02:02, HLA-B^∗^15:03, and HLA-C^∗^12:03 alleles with a global frequency of about 1.1, 0.63, and 3%, respectively, showed the highest affinity to bind against highly conserved SARS-CoV-2 peptides. Among them, HLA-B^∗^15:03 further shared with common coronaviruses, and the authors predicted that these HLA alleles can elicit cross-protective T-cell-mediated immunity (Nguyen et al., 2020). However, HLA-A^∗^25:01, HLA-B^∗^46:01, and HLA-C^∗^01:02 alleles with a global frequency of about 0.49%, 6.1%, and 7.8%, respectively, can interact with the least number of SARS-CoV-2 peptides (Nguyen et al., 2020). Similarly, an earlier study on probable SARS patients, health workers, and healthy Taiwanese people showed that HLA-B^∗^46:01 may favor SARS-CoV-1 infection (Lin et al., 2003). A separate study predicted that countries having HLA-A^∗^02:03 and HLA-A^∗^31:01 genotype may present epitopes from SARS-CoV-2 spike protein and have lower cumulative incidence per 1 million inhabitants, while HLA-A^∗^03:02 allele-bearing countries have a higher incidence of SARS-CoV-2 (Romero-López et al., 2020). Epitopes from SARS-CoV-2 including ORF1ab P4715L and S D614G mutations have been reported with high-efficiency binding capacity with MHC molecules including HLA-A^∗^11:01, HLA-A^∗^02:06, and HLA-B^∗^54:01 (Gonzalez-Galarza et al., 2020). Frequencies of these HLA alleles with a case per millions and the fatality rate (%) were statistically analyzed for 21 countries, including the worst COVID-19 affected Italy, Spain, England, United States, Brazil, France, and China, available in the Allele Frequency Net Database^3^ (Gonzalez-Galarza et al., 2020; Toyoshima et al., 2020). While frequencies of HLA-DQB1^∗^06 allele were significantly correlated with the SARS-CoV-2 infection in the United Kingdom (Poulton et al., 2020), the COViD-19 outbreak in Italy and China was positively correlated with allelic frequencies of HLA-B^∗^44 and HLA-C^∗^01 (Correale et al., 2020) and HLA-C^∗^07:29 and HLA-B^∗^15:27 (Wang et al., 2020b), respectively. However, although the positive correlation of HLA alleles with the outcome of COVID-19 in different geographical regions is reported in clinical, computational, and statistical models, more large-scale population studies are required to understand the regional disparity in terms of disease outcomes of COVID-19 due to HLA allelic variations.

**TABLE 2:** HLA reported to impact on COVID-19 incidence and case fatality around the globe.

**HLA with**		**Study population**	**References**
**Protecting**	**Non-protecting**		
HLA-A*02:02	–	Global (using database)	Nguyen et al., 2020
HLA-B*15:03	–		
HLA-C*12:03	–		
–	HLA-B*46:01	Taiwan	Lin et al., 2003
HLA-A*02:03	–	14 countries from America, Europe, and Asia	Gonzalez-Galarza et al., 2020
HLA-A*31:01	–		
–	HLA-A*03:02		
HLA-A*11:01	–	European region	Toyoshima et al., 2020
HLA-A*02:06	–		
HLA-B*54:01	–		
–	HLA-DQB1*06	United Kingdom	Poulton et al., 2020
–	HLA-B*44	Italy	Correale et al., 2020
–	HLA-C*01		
–	HLA-C*07:29	China	Wang J. et al., 2020
–	HLA-B*15:27		

### Immunodeficiency/Immunosuppression and the Host Response to COVID-19

Predisposition to infectious diseases is commonly expected in individuals with immunodeficiency/immunosuppression, which causes defects in humoral or cellular immunity or both (Abolhassani et al., 2018). However, it is still questionable whether immunodeficiency/immunosuppression is a predisposing or protective factor because very few studies have been done on immunodeficient/immunosuppressed subjects who developed COVID-19 (Babaha and Rezaei, 2020; Minotti et al., 2020). Although primary antibody deficiency negatively correlates with the severity of SARS-CoV-2 infection (Babaha and Rezaei, 2020), COVID-19 patients having agammaglobulinemia and B-cell deficiency showed milder clinical manifestation without the requirement of ICU or mechanical ventilation (Quinti et al., 2020). The intrinsic deficiency of B lymphocytes is supposed to prevent the development of inflammation in cytokine storm, which is associated with the morbidity of COVID-19 (Quinti et al., 2020; Saghazadeh and Rezaei, 2020; Yazdanpanah et al., 2020). By analyzing 110 COVID-19 patients with immunosuppression and immunodeficiency, Minotti et al. (2020) conclude that immunosuppressed patients are not significantly related to the overall figures to represent a favorable outcome as compared to other comorbidities. However, a study with a meta-analysis showed that immunosuppression and immunodeficiency increase 3.29- and 1.55-fold the risk of severity of COVID-19, respectively, although the statistical difference of their data were not significant (Gao et al., 2020). Nevertheless, their study was done with small sample size. Therefore, high-quality studies with larger and/or thorough data are necessary to draw a conclusive inference on the impact of immunosuppression and immunodeficiency on COVID-19.

### Regional Variance in Host Response to COVID-19

The morbidity and the mortality of COVID-19 significantly varied around the globe. Interestingly, most of the Asian countries have shown a different scenario than that of European, North American, and many other countries. The infection rate and severity of COVID-19 were comparatively much lower in Asian countries (Tanabe et al., 2020). The exact reason for regional variance in mortality and morbidity of COVID-19 is not yet clear. However, one of the highly speculated hypotheses is that the Bacillus Calmette-Guérin (BCG) vaccination program in Asian countries might have a protective role against SARS-CoV-2 infection because of the heterologous immune effects of BCG vaccine against unrelated pathogens other than *Mycobacterium tuberculosis* (Hirve et al., 2012; Miyasaka, 2020; Tanabe et al., 2020). BCG could have associated with the trained immunity process through manipulating epigenetics of immune cells to produce inflammatory cytokines such as IL-1, IL-6, and TNF to provide a beneficial effect against COVID-19 (Netea et al., 2020). However, different BCG strains may act differently because of their variable efficiency in inducing the production of several inflammatory cytokines (Davids et al., 2006; Wu et al., 2007; Hauer et al., 2020; Miyasaka, 2020). Recently, among Asian countries, the COVID-19 situation in India and Bangladesh has become severely deteriorated due to evolution of new variants, especially the fast-spreading delta variant.^4^

Another reason for the regional variance of the COVID-19 clinical manifestation could be the age ratio of the population of a country. It has been seen in several countries that children are significantly less infected by SARS-CoV-2 with mostly mild clinical manifestations (Hartford et al., 2020). Countries with an older age population may face relatively severe outcomes from SARS-CoV-2 than countries with a youthful population (Dowd et al., 2020; Imam et al., 2020; Njenga et al., 2020). As a casual correlation has been established, detailed studies are required to make an inference on the effect of age and BCG vaccination program on COVID-19 to understand the regional variance of host response. Furthermore, other factors such as lockdown program, social distancing, physical activities, ethnicity and food habit of the population, climatic condition, and presence of air pollution should be included in the regional variance of host response to SARS-CoV-2 (Baqui et al., 2020; Catherine Desoto, 2020; Frontera et al., 2020; Iqbal et al., 2020; Méndez-Arriaga, 2020).

## Host Response to SARS-CoV-2 Varies With Comorbidities

It is reported that about 40.80% of the total and 89.3% of the severe COVID-19 patients have comorbidity (Garg et al., 2020; Gold et al., 2020). The major comorbidities in COVID-19 patients are hypertension, cardiovascular diseases, diabetes, renal diseases, malignancy, and coinfections (Sanyaolu et al., 2020). Although the overall mortality rate of COVID-19 is low (1.4–2.3%) (Guan et al., 2020; Wu and Mcgoogan, 2020), comorbidities increase the severity of the disease and subsequent mortality (Table 3).

**TABLE 3:** Fatality rate in COVID-19 patients with different comorbidities.

**Comorbidities/disease**	**Fatality rate (%) in**				
	**NY, United States^a^**	**Brazil^b^**	**India^c^**	**Italy^d^**	**China^e^**
Hypertension	55.4	5.1	22.1	73.8	9.5
Diabetes	37.3	28.7	27.8	33.9	7.4
Coronary artery disease	12.4	35.1	6.2	30.1	7.3
Renal disease	11.0	5.9	2.27	20.2	0.7
COPD	8.3	8.2	13.6	13.7	7
Cancer	8.1	0.6	–	19.5	2

*^*a*^Sanyaolu et al. (2020).*

*^*b*^Pachiega et al. (2020).*

*^*c*^Majeed et al. (2020).*

*^*d*^Epidemiology for Public Health (2020).*

*^*e*^Ejaz et al. (2020).*

### Hypertension

Hypertension is a very common disease in the world, and almost 1.39 billion people are suffering from it (Mills et al., 2020). Most of the hypertensive patients are elderly people who fall at risk of death due to other complications if they are infected with SARS-CoV-2 (Schiffrin et al., 2020), and therefore, hypertension is being constantly reported all over the world as one of the major risk factors of COVID-19 (Wang D. et al., 2020; Yang J. et al., 2020; Savoia et al., 2021). Grasselli et al. (2020) reported that around 42% of 1,591 COVID-19 patients had hypertension. A baseline study from Wuhan of China suggested that about 27% of COVID-19 patients were associated with hypertension (Wu C. et al., 2020). Many reports have already showed the association of hypertension with the progression and severity of COVID-19 (Grasselli et al., 2020; Zhou F. et al., 2020), but still, many more studies are required to address this relation (Shibata et al., 2020). One study reported that hypertension increased the risk of both mortality and severity of COVID-19 ∼2.5-fold in patients of age 60 years and above (Lippi et al., 2020). The fatality rate of COVID-19 patients with hypertension has been reported for different countries (Table 3), and it is very high in Italy (73.8%), United States (55.4%), and India (22.1%). On the other hand, this rate is relatively less in Brazil (5.1%) and China (9.1%), and therefore, extensive large-scale studies in many more countries will provide the actual scenario (Ejaz et al., 2020; Pachiega et al., 2020). However, evidences attest that both systemic and pulmonary hypertension are risk factors for unfavorable progression of COVID-19 in patients with ARDS (Dai et al., 2019), multiple organ failure (Leoncini et al., 2013), and pneumonia (Chalmers et al., 2008). Hypertensive COVID-19 patients are more likely to have severe pneumonia, excessive inflammatory reactions, organ and tissue damage, and deterioration of the disease compared to COVID-19 patients without hypertension (Huang S. et al., 2020). Therefore, a comparison among COVID-19 patients with and without hypertension has made it clear that the coexistence of SARS-CoV-2 and hypertension increases the problem of unfavorable prognosis of COVID-19. As the death rate is dramatically high, hypertensive individuals need specific precautions such as maintaining social distance (1–2 m); wearing more protective accessories like goggles, gloves, FFP2 (N95), or FFP3 masks; and controlling blood pressure.

### Cardiovascular Diseases

While the median age of patients infected with SARS-CoV-2 is 56 years old, severe COVID-19 patients are middle-aged and elderly with preexisting comorbidities (Wang D. et al., 2020). In this age group, many chronic comorbidities including myocarditis, heart failure, cardiomyopathy, arrhythmia, hypertension, and diabetes mellitus start to develop (Karlamangla et al., 2007). A wide variety of cardiac abnormalities, namely, myocardial injury, myocarditis, pericarditis, arrhythmia, cardiac arrest, cardiomyopathy, heart failure, cardiogenic shock, and coagulation abnormalities, have been reported in COVID-19 patients with preexisting cardiovascular diseases (CVD), resulting in a large proportion of deaths (Table 3; Nishiga et al., 2020; Ssentongo et al., 2020; Zheng Y.Y. et al., 2020). Patients with hypertension and CVD have a higher expression of ACE2, which could be a potential reason for enhancing susceptibility of those patients to SARS-CoV-2 and a possible mechanistic pathway of severity of COVID-19 patient with CVD (Driggin et al., 2020). Besides, COVID-19 patients with CVD have hypercoagulability due to an increased level of D-dimer, which increases the risk of pulmonary embolism and, thus, hypoxia and heart failure (Li M. et al., 2020; Malik et al., 2021). Myocardial indicators including creatinine kinase, cardiac troponin I, and myoglobin are increased to varying degrees in COVID-19 patients, especially in ICU and severe patients (Wang D. et al., 2020; Malik et al., 2021). In patients with acute coronary syndrome, reduced cardiac function leading to myocardial ischemia might be a factor for severity of patients with COVID-19 and may lead to death (Zheng Y.Y. et al., 2020). The angiotensin II responsible for vasoconstriction, high blood pressure, and vascular remodeling (Tikellis and Thomas, 2012) increase significantly in COVID-19 patients, and it has been suggested that binding of SARS-CoV-2 to ACE2 causes a release of excessive angiotensin II through the renin–angiotensin system to increase heart loading, cardiomyocyte hypertrophy, and high blood pressure (Li M. et al., 2020). Therefore, SARS-CoV-2 infection affects cardiac-relevant biochemical pathways, such as the ACE2 signaling pathway, cardiac muscle integrity, fibrinogen pathways, and redox homeostasis; induces a break in plaque associated with the stent; and, finally, aggravates a myocardial injury and dysfunction (Bansal, 2020; Liaqat et al., 2021).

### Diabetes Mellitus

Patients with diabetes mellitus (DM) who develop COVID-19 have been observed to have a worse prognosis and increased mortality in most studies (Table 3; Guan et al., 2020; Sanyaolu et al., 2020; Wu Y.C. et al., 2020). A study with 1,099 confirmed COVID-19 cases showed that DM was prevalent (16.2%) in patients with severe disease (Guan et al., 2020), and an analysis of 72,314 confirmed COVID-19 cases from the Chinese Center for Disease Control and Prevention revealed higher morbidity (7.3%) with DM (Wu Y.C. et al., 2020). Another study with 1,122 COVID-19 patients from 88 centers across the United States found DM to be associated with more than four-fold increase in mortality (Bode et al., 2020). A single-centered, independent study showed that COVID-19 patients with DM exhibited severe inflammatory response having intensified multiple pathophysiological events associated with cytokine storm, venous thromboembolism, cardiac and kidney failure, and liver function damage (Malik et al., 2021). Thus, most of the studies showed that DM associated as a distinctive comorbidity increases the morbidity and mortality of COVID-19 patients (Guan et al., 2020; Sanyaolu et al., 2020; Wu Y.C. et al., 2020; Malik et al., 2021).

The immune system, particularly the innate immunity, remains compromised in uncontrolled DM patients and may allow unrestricted replication of the SARS-CoV-2 (Geerlings and Hoepelman, 1999; Angelidi et al., 2020). Increased SARS-CoV-2 replication in DM patients might be due to an increase in furin, a membrane-associated protease involved in the entry of coronaviruses into the cell (Fernandez et al., 2018). Furthermore, numerous factors such as hyperglycemia, altered production of cytokine, impaired T-cell-mediated immune response, impaired natural killer cell activity, defects in complement action, inhibition of neutrophil chemotaxis, ineffective viral clearance, and phagocytic cell dysfunction may contribute to immune dysfunction in DM individuals and increase their severity to COVID-19 (Cure and Cumhur Cure, 2020; Nyambuya et al., 2020; Han et al., 2021). It is suggested that the ACE2, which are expressed on many cells surface as mentioned above, may play a vital role in the severity of COVID-19 infection in DM individuals (Huang I. et al., 2020; Pal and Bhansali, 2020). Besides, individuals with DM have been shown to have elevated levels of proinflammatory cytokine, especially IL-1, IL-6, and TNF-α, and different markers such as C-reactive protein, D-dimer, and fibrinogen, which may further prolong cytokine storms and lead to severe illness in DM individuals with COVID-19 infection (Huang I. et al., 2020; Han et al., 2021; Malik et al., 2021). However, it is still elusive whether DM itself certainly increases morbidity and mortality of COVID-19 patients or cardiovascular and renal comorbidities that are commonly associated with DM are the foremost factors involved. Nevertheless, DM independently contributes to mortality and morbidity in patients with SARS-CoV-1 (Yang et al., 2006). Based on different studies (Muniyappa and Gubbi, 2020; Pal and Bhansali, 2020), multiple mechanisms can be proposed to be involved in the increased susceptibility of individuals with pre-existing DM to COVID-19: (i) greater affinity to SARS-CoV-2 for cellular binding and entry; (ii) reduced viral clearance; (iii) reduced T-cell function; (iv) co-existence of cardiovascular and renal diseases; and (v) susceptibility to cytokine storm and hyper-inflammation.

### Chronic Obstructive Pulmonary Disease

The susceptibility of the patients with chronic obstructive pulmonary disease (COPD) to SARS-CoV-2 is given priority because respiratory failure, vascular thrombosis, diffuse alveolar damage, and pulmonary edema are usually the common scenario in severely affected COVID-19 patients (Ackermann et al., 2020). COPD is common in 10% of COVID-19 patients over the age of 45 (Singh et al., 2019), and the fatality rate is 7–13% in different countries (Table 3). The CDC, United States, claims that approximately one third of the hospitalized COVID-19 patients somehow experienced prior respiratory complications such as COPD (Sikjaer et al., 2018). A recent epidemiological study suggested that COPD was prevalent in 1.1–38% of COVID-19 patients (Leung et al., 2020). During the early days of COVID-19 infections in China, it had been reported that around 13.6% of adults over 40 years of age were SARS-CoV-2-infected patients with COPD complications (Leung et al., 2020). Different studies show that the prevalence of COPD in hospitalized COVID-19 patients is 2.4–14% in New York (Kuno et al., 2020; Palaiodimos et al., 2020; Richardson S. et al., 2020), 5.6–9.2% in Italy (Cecconi et al., 2020; Inciardi et al., 2020), and 17.7% in the United Kingdom (Leung et al., 2020). However, a higher level (38%) of COPD association with COVID-19 patients was reported in 48 ICU-admitted patients in Spain (Barrasa et al., 2020), though it was only 4% in 1,591 ICU-admitted patients from Italy (Bhatraju et al., 2020). Although different studies suggested COPD prevalence among SARS-CoV-2-infected patients, it was still lower than other comorbidities, such as diabetes or hypertension.

The exact reason behind the COPD prevalence among SARS-CoV-2-infected patients is still unanswered; however, a few possibilities could hypothesize COPD as the risk factor of severe COVID-19 progression. COPD patients with COVID-19 are usually characterized by the higher level of ACE2 expression in the respiratory tract (Leung et al., 2020; Milne et al., 2020), which in turn might have increased the susceptibility of the host to SARS-CoV-2 infection. Nicotine exposure through smoking has also been reported by several studies as the enhancer of ACE2 expression (Cai G. et al., 2020; Li et al., 2020b; Zhang H. et al., 2020). Besides, COPD patients experience a higher level of anxiety and depression in the renin–angiotensin–aldosterone system, which could be associated with up-regulation of angiotensin II and ACE (Toru et al., 2015). All these phenomena collectively may lead to increased pulmonary edema or pulmonary hypertension following SARS-CoV-2 infection. Pulmonary edema rapidly developing in COVID-19 patients with COPD is considered as one of the reasons for death (Mariajoseph-Antony et al., 2020). Transporter proteins such aquaporins, which are in the check point of cellular inflammations and pulmonary edema, may be considered as potential drug targets to modulate their expression levels to mitigate the inflammation-induced comorbidity in COVID-19 patients (Mariajoseph-Antony et al., 2020; Azad et al., 2021). Moreover, a medication strategy of COPD patients had also been claimed to be associated with the risk of COVID-19. A study reported that around 44.8% of COVID-19 patients were found to have COPD medication with inhaled corticosteroids, whereas only 18.3% of COVID-19 patients associated with COPD were without corticosteroid medication (Attaway et al., 2020). As there are still different questions regarding the positive correlation between COPD and COVID-19, research should be continued to break down the true figure of COVID-19 progression in COPD patients. However, proper management and mitigation attempts with correct inhaler-based medication of COPD patients could reduce the risk of SARS-CoV-2 infection.

### Renal Disorder

Kidney disorder is one of the most frequent comorbidities in patients infected with SARS-CoV-2, especially in severely ill patients (Chen N. et al., 2020; Wang D. et al., 2020; Yang X. et al., 2020). AKI increases the susceptibility of patients to SARS-CoV-2, and the prevalence of AKI has been reported as high as 68% in critically ill COVID-19 patients in New York (Naicker et al., 2020). The mortality rate of COVID-19 patients with AKI is more than those without AKI (Table 3). Sepsis with direct cellular damage or cytokine storm syndrome caused by the SARS-CoV-2 has been hypothesized as the plausible mechanism. SARS-CoV-2 may induce cytopathic effects on kidney cells through ACE2 receptor expressed in proximal tubules, afferent arterioles, and loop of Henle (Naicker et al., 2020; Rudiansyah et al., 2020). The expression level of ACE2 in urinary tracts has been reported almost 100-fold higher than that in respiratory tracts, which in turn facilitates efficient entrance of SARS-CoV-2 to kidney cells to cause kidney diseases (Devaux et al., 2020). Acute tubular necrosis may occur due to SARS-CoV-2 invasion to kidney tubules (Danser et al., 2020; Qian et al., 2020). The elevation of D-dimer and prolongation of prothrombin time, activated partial thromboplastin time, and international normalized ratio observed in COVID-19 patients may induce disseminated intravascular coagulopathy (Batlle et al., 2020). They have pleiotropic effects for production of a higher level of some inflammatory mediators including IL-1, IL-6, TNF-α, and INF-α. In critically ill COVID-19 patients, the cytokine storm syndrome is hypothesized to block the vascularization of the kidney to cause the acute renal failure (Harenberg and Favaloro, 2020; Levi, 2020; Raza et al., 2020).

### Cancer

Cancer, which is the top in the causes of death, has the highest incidence in Asia, Europe, and North America (Bray et al., 2018). Cancer patients having COVID-19 more likely need admission to ICU with mechanical ventilation compared to COVID-19 patients without cancer. If cancer patients are infected by SARS-CoV-2, the cancer treatment becomes delayed until they recover from the virus (Shankar et al., 2020). Chemotherapy and some targeted therapies for cancer can cause neutropenia, a temporary depletion of white blood cells that fight against infection (Akula et al., 2020). Risk factors include not only age but also the kind of cancer, the stage, and the treatment (Liang et al., 2020; Yu J. et al., 2020). Blood cancers such as leukemia, lymphoma, and myeloma reduce patients’ natural defenses and make them susceptible to precarious infections (Moujaess et al., 2020). Age, sex, and tumor type revealed that older COVID-19 patients with hematologic cancers (leukemia, lymphoma, and multiple myeloma) have more severe COVID-19 trajectory compared to patients with solid organ tumors (Lee et al., 2020). In addition, lung cancer makes COVID-19 patients more vulnerable because of reduced lung function. Cancer patients with blood or lung malignancies that have spread throughout the body have a three-fold higher risk of death or other severe complications from COVID-19 compared to those without cancer (Addeo and Friedlaender, 2020). However, although it is severe, COVID-19 accounted for a minority of overall lung cancer deaths during the pandemic (Table 3).

### Coinfections

Up to 50% of COVID-19 patients are exposed to viral and bacterial respiratory pathogens (Kim et al., 2020b; Lehmann et al., 2020; Li Z. et al., 2020; Lin et al., 2020; Wee et al., 2020; Wu Q. et al., 2020). Surprisingly, a higher rate of coinfection has been reported in children, although the sample size was small (Wu Q. et al., 2020). The common respiratory pathogens that coinfected COVID-19 patients are respiratory syncytial virus, human metapneumovirus, human parainfluenza virus, influenza A, *Mycoplasma pneumoniae*, *Streptococcus pneumoniae*, Epstein–Barr virus, non-SARS-CoV-2 Coronaviridae, cytomegalovirus, rhinovirus, and enterovirus (Chen F.L. et al., 2020; D’Ardes et al., 2020; Fan et al., 2020; Jiang et al., 2020b; Nieto-Moro et al., 2020; Toombs et al., 2020; Touzard-Romo et al., 2020). The impact of coinfection on the clinical manifestations of COVID-19 has created a dilemma. COVID-19 patients with coinfections may experience difficulties in breathing and develop ARDS and shock (Khaddour et al., 2020). No amplitude in morbidity or mortality in COVID-19 patients for the coinfection reported by Wee et al. (2020) contradicts with the findings of Lehmann et al. (2020) and Li Z. et al. (2020). The latter two studies observed a higher frequency of coinfection among ICU patients. The lymphocyte and platelet counts are also comparable between COVID-19 patients with and without coinfections (Li Z. et al., 2020). Coinfections of SARS-CoV-2 with bacterial respiratory pathogens like *S. pneumoniae* (Nieto-Moro et al., 2020; Toombs et al., 2020), *Klebsiella pneumoniae* (Chen W.C. et al., 2020), and *M. pneumoniae* (Fan et al., 2020) might have caused severity in COVID-19 patients (Ou X. et al., 2020). Therefore, the opportunistic infection may worsen the clinical outcome of COVID-19 (De Freitas Ferreira et al., 2020).

Coinfection of SARS-CoV-2 with influenza virus was firstly reported from China, then many more countries like the United States, Germany, Turkey, Japan, Iran, Italy, Taiwan, and Spain were included in the list (Azekawa et al., 2020; Cuadrado-Payán et al., 2020; D’Abramo et al., 2020; Hashemi et al., 2020; Huang B.R. et al., 2020; Kondo et al., 2020; Nowak et al., 2020; Ozaras et al., 2020; Wehl et al., 2020; Wu X. et al., 2020). While low coinfection rate of SARS-CoV-2 with influenza was reported in many studies (Kim et al., 2020b; Nowak et al., 2020; Ozaras et al., 2020; Zheng X. et al., 2020), an opposite observation of very high coinfection rate of SARS-CoV-2 with influenza was reported (Yue et al., 2020). However, the rate of coinfection of SARS-CoV-2 with influenza might be affected by (i) the flu season (Wehl et al., 2020), (ii) the restrictions and social distancing to constrain COVID-19 outbreak (Sakamoto et al., 2020), and (iii) different geographic regions (Azekawa et al., 2020; Hashemi et al., 2020).

An important perspective of COVID-19 is its impact on patients with tuberculosis (TB), as TB is a global burden causing huge deaths worldwide (yearly around 1.5 million deaths) (WHO, 2020d). Unfortunately, very few have been known about the coinfection of SARS-CoV-2 in TB patients. Tadolini et al. (2020) reported 49 COVID-19 cases with old/active TB. A high mortality rate (6/49, 12.3%) was observed in this study. On the other hand, Stochino et al. (2020) stated mild clinical manifestation (5% fatality rate) of 20 COVID-19 cases among 24 active TB-diagnosed patients. Therefore, larger studies are required to get a conclusive hint regarding this issue.

## Host Response Toward the Treatment Strategy for COVID-19

Although there are no newly developed specific drugs against COVID-19, several supportive treatments mentioned below are given to COVID-19 patients.

### Drug Repurposing Against COVID-19

To tackle the mortality of the COVID-19 pandemic, various approved drugs with known safety profiles were assessed against SARS-CoV-2 (Poduri et al., 2020). The FDA approved remdesivir, a nucleoside analog that inhibits viral RNA-dependent RNA polymerase, for emergency use on May 1, 2020 (Agostini et al., 2018; Sheahan et al., 2020). Remdesivir has a better efficacy and lower toxic side effects as an anti-SARS-CoV-2 on Vero E6 cells (Wang M. et al., 2020) and shorten the recovery time in hospitalized COVID-19 patients (Beigel et al., 2020). Favipiravir, an RNA-dependent RNA polymerase inhibitor, currently used in Japan for the treatment of influenza infections, has been shown by clinical trial to be useful against SARS-CoV-2 with the therapeutic response especially with a significantly shorter median time to viral clearance, hospital stay, and the need for mechanical ventilation (Cai Q. et al., 2020; Chen C. et al., 2020; Ivashchenko et al., 2020; Chen et al., 2021; Dabbous et al., 2021; Joshi et al., 2021; Udwadia et al., 2021). Baritinicib, another FDA-approved drug for the treatment of rheumatoid arthritis, might be useful for the treatment of COVID-19 and urged for a randomized clinical trial (Cantini et al., 2020a, b). However, long-term administration of baritinicib may enhance coinfection (Favalli et al., 2020; Praveen et al., 2020; Richardson P.J. et al., 2020).

An *in vitro* study showed that ivermectin, an FDA-approved anti-parasitic drug, is effective against the SARS-CoV2, but doubt was raised regarding the dose for the effectiveness from pharmacokinetic aspects (Momekov and Momekova, 2020; López-Medina et al., 2021; Peña-Silva et al., 2021). However, several studies suggest ivermectin to improve clinical recovery and prognostic laboratory parameters and decrease mortality with mild to severe COVID-19 (Khan et al., 2020; Rajter et al., 2020; Ahmed et al., 2021; Okumuş et al., 2021; Pott-Junior et al., 2021). Very recently, a multimodal technology and screening in Huh7 cells revealed that four drugs (auranofin, azelastine, digoxin, and vinblastine) could be repurposed as novel treatments for COVID-19 (Morselli Gysi et al., 2021).

### Interleukin-6 Inhibitors

An elevated level of IL-6, a key proinflammatory cytokine produced by macrophages, is considered for mediating inflammation in COVID-19, increasing severity and fatality and dysregulation of the host response to SARS-CoV-2 (Chen X. et al., 2020; Gubernatorova et al., 2020; Jones and Hunter, 2021). Therefore, IL-6 receptors appear to be a promising therapeutic target as IL-6 receptor blockers are reported to have lower mortality among various patient groups having a diverse level of COVID-19 severity (Rubin et al., 2021). Anti-IL-6 receptor monoclonal antibodies (e.g., sarilumab and tocilizumab) and anti-IL-6 monoclonal antibodies (i.e., siltuximab), approved by the FDA, are now being widely used in severe COVID-19 patients as these are effective based on several small studies and case reports (Michot et al., 2020; Remap-Cap Investigators, 2021).

A study involving severely and critically ill COVID-19 patients demonstrated that several doses of tocilizumab might be an effective treatment option in COVID-19 patients with a risk of cytokine storms (Luo et al., 2020). Xu et al. (2020) reported that 21 severe or critical COVID-19 patients were recovered and discharged within 2 weeks after treatment with tocilizumab without adverse drug effects. However, results from another randomized control trial involving hospitalized patients with severe COVID-19 did not result in better clinical status or lower mortality than placebo at 28 days (Rosas et al., 2021). A clinical case series study showed that sarilumab had promising potential to improve the symptoms by reducing oxygen demand in eight hospitalized COVID-19 patients (Benucci et al., 2020). However, a randomized, double-blind placebo control multinational phase 3 trial of sarilumab did not show significant efficacy (Lescure et al., 2021). A cohort-controlled study among 30 patients in Italy reported that siltuximab treatment declined the mortality rate of patients requiring ventilation significantly (Gritti et al., 2021). However, results from different studies suggest that the treatment of critically ill COVID-19 patients only with the inhibitory modulators of the IL-6 pathway may not be enough.

### Combination Treatment for COVID-19

Combination therapies, which not only targets the virus but also host functions like boosting host immune response, are a promising approach for treating COVID-19. To date, 608 combination treatment studies (combinations of antivirals, antibodies, herbal, ayurvedic, and traditional medicines) have been registered in the US clinical trial database.^5^ One of the preliminary successes showed that the combination of interferon beta-1b, lopinavir–ritonavir (protease inhibitor), and ribavirin (nucleoside analog) reduced the hospital stay 5 days (Hung et al., 2020). Remdesivir was tested in combination with interferon beta-1a (clinical trial ID: NCT04492475) and hyperimmune intravenous immunoglobulin (clinical trial ID: NCT04546581) for reducing the recovery time for COVID-19 patients. On November 19, 2020, FDA approved the combination use of baricitinib and remdesivir to treat suspected or hospitalized COVD-19 patients. The combination use of baricitinib and remdesivir has been reported to reduce the recovery time at least 8 days compared to the placebo control and to accelerate the improvement of clinical status in COVID-19 patients (Kalil et al., 2020). The combination of monoclonal antibodies, bamlanivimab and etesevimab, was also approved for emergency use against COVID-19 by the FDA on February 09, 2021. The clinical studies and approval of combination treatments against COVID-19 indicate that the effectiveness of combination therapy works differently in different groups of patients due to the versatile host–virus dynamics.

### COVID-19 Vaccines Against SARS-CoV-2 Variants

Since the turbulence of the ongoing pandemic, researchers around the world are trying their level best for vaccine development against COVID-19 considering different strategies including (i) mRNA-based vaccines, (ii) viral vector vaccines, (iii) inactivated viral vaccines, and (iv) recombinant subunit vaccines (reviewed by Pormohammad et al., 2021). The efficacy and safety of the vaccines under randomized clinical trials have been discussed elsewhere through systematic reviews and meta-analysis (Mcdonald et al., 2021; Pormohammad et al., 2021). A number of vaccines against COVID-19, namely, BNT162b2 (Pfizer), mRNA-1273 (Moderna), AZD1222/Covishield/ChAdOx1 (AstraZeneca), Ad26.COV2.S (Janssen/Johnson & Johnson), and SARS-CoV-2 Vaccine (Sinopharm/BIBP and Sinovac), have already been approved by the WHO.^6^ It has been reported that the number of new SARS-CoV-2 infections against symptomatic and high viral burden infections is largely reduced following the second dose of vaccination with BNT162b2, mRNA-1273, and ChAdOx1 (Keehner et al., 2021; Pritchard et al., 2021). However, an individual study of each vaccine against different VOC of SARS-CoV-2 shows the vaccine effectiveness (VE) ranging 42–100% (Table 4). Nevertheless, the durability of immune response of these vaccines is not yet established. While one study done in Qatar shows almost similar VE of BNT162b2 against B.1.1.7 and B.1.351 variants (Abu-Raddad et al., 2021), another study done in Finland reports at least five-fold lower VE against B.1.351 variant compared to that against B.1.1.7 variant (Jalkanen et al., 2021). This variance in VE of the same vaccine against the same variant may be associated with the host factors aforementioned. However, these two studies support the notion that following completion of the second dose of vaccination, all the approved vaccines may induce cross-neutralization of at least some of the circulating SARS-CoV-2 variants. It has been showed that the single-shot Ad26.COV2.S is effective against the rapidly spreading Delta variant and other VOC (Jongeneelen et al., 2021). Although the VE of BNT162b2 and ChAdOx1 has been compared against Alpha and Delta variants showing similar efficacy (Stowe et al., 2021), there is no single study comparing the VE of all vaccines so far approved by the WHO against all VOC and VOI. Therefore, it is necessary to compare the VE of all vaccines by applying to subjects of different regions/countries against all VOC and VOI including their durability of immune response.

**TABLE 4:** Effectiveness of approved vaccines against different SARS-CoV-2 variants.

**Name of vaccine**	**Active against variants**	**Vaccine effectiveness (%)**	**Days after vaccination**	**References**
mRNA-1273 (Moderna)	Wild-type	94.1%	14 days after the second dose	Baden et al., 2020
	Alpha B.1.1.7	92–100%	≥7–14 days after the second dose	Chemaitelly et al., 2021; Nasreen et al., 2021; Noh et al., 2021
	Beta B.1.351	96.4%		
	Gamma P.1	77%	≥14 days after first dose	Nasreen et al., 2021
	Delta B.1.617.2	72%		
BNT162b2 (Pfizer)	Wild-type	95%	21 days after the second dose	Polack et al., 2020
	Alpha B.1.1.7	87–97.4%	≥14 days after the second dose	Abu-Raddad et al., 2021; Sheikh et al., 2021; Wall et al., 2021
	Beta B.1.351	72.1–97.4%		
	Delta B.1.617.2	79–88%	≥14 days after the second dose	Lopez Bernal et al., 2021; Sheikh et al., 2021; Wall et al., 2021
Ad26.COV2.S (JNJ-78436735)	Wild-type	66.3%	≥14 days after getting vaccinated	Sadoff et al., 2021
	Delta B.1.617.2	1.6-fold reduction/lab data by pseudovirus assay (60%)	71 days after single-dose vaccination	Jongeneelen et al., 2021
AZD1222/Covishield/ChAdOx1 (AstraZeneca)	Wild-type	66.7%	≥14 days after the second dose	Voysey et al., 2021
	Alpha B.1.1.7	66–73%	After second dose of vaccination	Bernal et al., 2021; Lopez Bernal et al., 2021; Sheikh et al., 2021
	Delta B.1.617.2	60–67%		
SARS-CoV-2 Vaccine (Sinopharm/BIBP)	Wild-type	72.8–86%	14 days after the second dose	Al Kaabi et al., 2021
SARS-CoV-2 Vaccine (Sinovac)	Wild-type	66–90%	≥14 days after the second dose	Jara et al., 2021; Tanriover et al., 2021
	Gamma P.1	42%	≥14 days after the second dose	Ranzani et al., 2021

## Conclusion and Future Perspectives

The common symptoms of COVID-19 are fever, cough, sore throat, shortness of breath, body pain, fatigue, headache and weakness, chest pain, loss of test or smell, impaired vision, dizziness, impaired consciousness, stroke, nerve pain, etc. Importantly, the disease is manifested as mild to moderate in almost 90% cases, and only 10% of cases need hospitalization (Bialek et al., 2020; Wang D. et al., 2020). The differential incursion of SARS-CoV-2 among different people is due to the variability of factors from the pathogen itself as well as from the host and the environment. In this review, we indeed looked at the dynamics of host response to SARS-CoV-2 in context of the viral-to-host factors.

Despite having similar clinical manifestations like SARS-CoV-1 and MERS-CoV, SARS-CoV-2 and its VOC have higher transmissibility, which ultimately leads to the ongoing COVID-19 pandemic. From literatures, it is clear that people of different races, regions, and ages with or without comorbidity are affected differently by SARS-CoV-2 with a wide range of morbidity and mortality. So, the current challenge for the researchers is to understand the molecular insights of the dynamics of host response to SARS-CoV-2 from different perspectives to develop effective strategies to combat the ongoing and any future outbreak.

Substantial data are available on the genome sequence and some major proteins, like spike glycoproteins, and on the differences among variants of SARS-CoV-2. However, more studies are necessary to decipher the roles of small accessory proteins from *ORF3*, *ORF6*, *ORF7*, *ORF8*, and *OFR10* in the infection dynamics and disease progression. Apart from the viral diversity, host response to the SARS-CoV-2 infection is dynamic and varies from person to person, i.e., people with the same viral load have different clinical manifestations, from asymptomatic to critically hospitalized, due to their age, sex, health conditions, presence of comorbidities, and geo-cultural and environmental factors, which urges to identify group-wise better intervention strategies to combat the disease. COVID-19 patients already suffering from comorbidities aforementioned become severe after SARS-CoV-2 infection, having a higher death rate, although the severity and the death rate differ from country to country (Table 3). Besides, coinfection with bacteria and virus can promote some clinical manifestations like ARDS and shock, and coinfected COVID-19 patients more likely require specialized treatment in the hospital.

Apart from these, genetic factors of the host may play a vital role to determine the severity and manifestation of the COVID-19. People having a certain blood group have shown different outcomes of COVID-19 (“O” with lower risk, whereas “A” and “AB” were associated with higher risk), and HLA genotypes (HLA-A^∗^02:02, HLA-B^∗^15:03, HLA-C^∗^12:03, HLA-A^∗^02:03, HLA-A^∗^31:01, HLA-A^∗^11:01, HLA-A^∗^02:06, and HLA-B^∗^54:01) have protecting immunity against COVID-19 (Table 2). Although these reports are not reflecting the overall scenario, they shed important lights on the dynamics of host response to SARS-CoV-2 infection. Besides, the dynamics of host response toward vaccine and treatment strategies against SARS-CoV-2 including their variants is associated with the viral and host factors.

Due to very high human-to-human transmissibility of COVID-19, a conclusive understanding of the disease dynamics is required to combat against it. Almost all studies have been done separately with a very small number of cases from a particular area/region of a country. Besides, developing counties have a fragile health system and the patient registry might be poor. Currently, disease control agencies from different countries have different policies and strategies to intervene with the pandemic and a lack of coordination, and therefore, unified data is not coming to understand the major trends of the disease progression globally in different groups. To do so, it is a challenge to adopt initiatives to conduct systematic longitudinal studies covering peoples of all groups from all races and geographical regions having a wide variety of systemic disorders. Therefore, international agencies like the WHO can coordinate this for better understanding of the disease dynamics, which will ultimately help to develop a sustainable framework to make data-driven decisions for participating countries to local authorities. Furthermore, this type of study is necessary to determine the VE of all approved vaccines against all VOC and VOI and the durability of their immune response.

## Author Contributions

AKA, MH, MMH, and AH made the concept and design. AH, MMH, MH, AKA, SML, KFA, and TR did the partial manuscript writing. AH, MMH, SML, KFA, and AKA performed data collection and analysis and figure preparation. PAC did literature collection and critical review. AKA performed data interpretation, compilation, and supervision and edited the whole manuscript. All authors contributed to the article and approved the submitted version.

## Conflict of Interest

The authors declare that the research was conducted in the absence of any commercial or financial relationships that could be construed as a potential conflict of interest.

## Publisher’s Note

All claims expressed in this article are solely those of the authors and do not necessarily represent those of their affiliated organizations, or those of the publisher, the editors and the reviewers. Any product that may be evaluated in this article, or claim that may be made by its manufacturer, is not guaranteed or endorsed by the publisher.

## Footnotes

1
https://extranet.who.int/pqweb/sites/default/files/documents/Status_COVID_VAX_02July2021.pdf

2
https://www.who.int/publications/m/item/weekly-epidemiological-update-on-covid-19—29-june-2021

3
http://www.allelefrequencies.net/

4
https://www.who.int/publications/m/item/weekly-epidemiological-update-on-covid-19—13-july-2021

5
https://clinicaltrials.gov/

6
https://extranet.who.int/pqweb/sites/default/files/documents/Status_COVID_VAX_02July2021.pdf
